# Real-World First-Line Treatment Patterns and Outcomes in Hormone Receptor-Positive Advanced Breast Cancer Patients: A Multicenter, Retrospective Study in China

**DOI:** 10.3389/fonc.2022.829693

**Published:** 2022-03-03

**Authors:** Zhanhong Chen, Quchang Ouyang, Yongsheng Wang, Junsheng Wang, Haixue Wang, Xiaohong Wu, Peili Zhang, Jian Huang, Yabing Zheng, Wenming Cao, Xiying Shao, Ning Xie, Can Tian, Hao Liang, Cailing Wang, Ying Zhang, Dianquan Ren, Xiaojia Wang

**Affiliations:** ^1^ Department of Breast Medical Oncology, Cancer Hospital of the University of Chinese Academy of Sciences (Zhejiang Cancer Hospital), Hangzhou, China; ^2^ Department of Breast Cancer, Hunan Cancer Hospital, Changsha, China; ^3^ Department of Breast Surgery, Shandong Cancer Hospital, Jinan, China; ^4^ Department of Breast Cancer, Anyang Tumor Hospital, Anyang, China; ^5^ Department of Breast Surgery, Anyang Tumor Hospital, Anyang, China; ^6^ Department of Breast Cancer, Affiliated Hospital of Jiangnan University, Wuxi, China; ^7^ Department of Breast Surgery, Baotou Cancer Hospital, Baotou, China

**Keywords:** advanced breast cancer, hormone receptor-positive, first-line treatment, outcomes, real-world study

## Abstract

**Background:**

Recent data on first-line treatment patterns administered to hormone receptor-positive (HR+) advanced breast cancer (ABC) patients in the real-world setting are limited. This study aimed to report the first-line treatment patterns and outcomes of HR+ ABC patients in China.

**Methods:**

This was a multicenter, noninterventional study. Eligible patients were cytologically or histologically confirmed to have HR+ ABC with ≥2 complete medical records and received first-line therapies between January 2015 and June 2019. Treatment patterns and outcomes were extracted from structured or unstructured electronic medical records. Progression-free survival (PFS) was analyzed with the Kaplan-Meier method.

**Results:**

In total, 1072 patients with HR+ ABC were enrolled at 6 treatment sites: 327 human epidermal growth factor receptor 2-positive (HER2+) patients, 696 HER2-negative (HER2-) patients and 49 HER2-unknown patients. Overall, 62.41% of patients received first-line chemotherapy (CT), 21.08% received targeted therapy (TT) and 15.49% received endocrine therapy (ET). For HR+/HER2+ patients, 65.14% received TT, 28.44% received CT, and 5.81% received ET. Compared with patients who received TT, patients who received CT alone, had a significantly worse median PFS (adjusted hazard ratio [HR] =2.59, 95% confidence interval [CI], 1.64-4.10, p<0.001). For HR+/HER2- patients, 77.01% received CT, 20.69% received ET and 1.15% received TT. Compared with patients who received ET, patients who received CT with maintenance therapy had a significantly prolonged median PFS (adjusted HR =0.57, 95% CI, 0.44-0.76, p<0.001). Among HR+/HER2- patients who received CT with maintenance treatment, those with maintenance ET had a longer median PFS than those with maintenance CT, but the difference was not significant (adjusted HR=0.92, 95% CI, 0.64-1.33, p=0.66).

**Conclusions:**

This real-world study demonstrates that CT remains the mainstream first-line treatment option for HR+ patients in China. Among patients with HR+/HER2+ ABC, the majority received first-line TT and experienced a PFS benefit. A high percentage of HR+/HER2- patients received CT as first-line therapy in clinical practice. PFS benefit was significantly longer in patients who received CT with maintenance therapy. Moreover, there was no obvious difference in PFS between maintenance ET and CT. Maintenance ET may be a better choice considering its lower toxicity and better quality of life.

## Introduction

Breast cancer is a common cancer, accounting for approximately 30% of female cancers, and has a mortality-to-incidence ratio of 15% (1). It is also the leading cause of cancer-related death in Chinese women, with an incidence rate of 19.2% and a mortality rate of 9.1% (2). Nearly two-thirds of patients with breast cancer in China are diagnosed with advanced disease (3, 4). Advanced breast cancer (ABC) comprises locally advanced breast cancer (stages IIIB/C) and metastatic breast cancer (stage IV); common sites of spread are bone, the lung and the liver (5). An estimated 5–10% of all breast cancer patients have metastatic disease at initial presentation, whereas approximately 30% of patients diagnosed with early-stage disease will progress to metastatic disease (6, 7). ABC is a treatable but virtually incurable disease, with metastases being the cause of death in almost all patients, a median overall survival period of 2–3 years and a 5-year survival rate of approximately 25% (5, 8, 9).

ABC therapies are formulated according to molecular subtypes. At the molecular level, the molecular features of ABC include the activation of human epidermal growth factor receptor 2 (HER2) and hormone receptor (HR, including estrogen receptor [ER] and progesterone receptor [PR]) expression (8). HR-positive (HR+) breast cancer is the most common phenotype, with the proportion reaching approximately 70–80% (10). For HR+/HER2-positive (HER2+) patients, the preferred first-line treatment option is anti-HER2 agents combined with chemotherapy (CT) agents (5, 11). For HR+/HER2-negative (HER2-) patients, clinical guidelines recommend first-line endocrine therapy (ET) with or without targeted therapy unless they are experiencing visceral crises and/or endocrine resistance is known or suspected (5). ET is supported by data showing a therapeutic benefit with less toxicity and better quality of life. First-line ET can be an aromatase inhibitor (AI), fulvestrant or tamoxifen, depending on the previous therapies and tumor progression (8, 12). If no more ET options or there is rapid progression or visceral crisis, CT is also recommended (5, 13). It is generally thought that CT is associated with a greater and earlier tumor response, especially in cases of a high disease burden (14). However, for women with HR+/HER2- ABC, which specific patients are suitable for CT or ET as first-line treatment remains unclear; to date, no randomized clinical trials have answered this question (14). Moreover, recent data on first-line treatment patterns and outcomes administered to HR+ ABC patients in the real-world setting are limited.

Therefore, our analysis of this multicenter, noninterventional study described first-line therapies and clinical outcomes, and analyzed the association between patient- and disease-related factors and outcomes in patients with HR+ ABC in a real-world setting in China.

## Methods

### Study Design

This was a multicenter, noninterventional, retrospective study conducted from January 2015 to June 2019. A total of 6 tertiary first-class hospitals were involved in this research (**Supplementary Table 1**). The primary objectives of this study were to describe the first-line treatment patterns and clinical outcomes of HR+ ABC patients. The secondary objectives were to explore associations between demographic and clinical factors and outcomes. According to HER2 status, the patients were categorized into the HER2+, HER2-, or HER2-unknown groups. ABC in this study included locally advanced breast cancer (stages IIIB/C) and metastatic breast cancer (stage IV). ER-or PR-positive was defined as the cutoff point of 1% of stained cells or recorded as positive by physicians in medical records. Any ER- and/or PR-positive was regarded as HR+. HER2+ was defined as either an immunohistochemistry (IHC) score 3+ or an IHC score 2+ and positive fluorescence *in situ* hybridization (FISH) or recorded as positive by physicians in medical records. First-line treatment was defined as initial therapy received for ABC up to first progression or therapy change. CT alone was defined as the use of CT agents only. CT with maintenance therapy refers to the continuation of CT agents and/or endocrine agents and/or anti-HER2 agents after discontinuation of CT agents (5). ET was defined as the use of endocrine agents only. Targeted therapy (TT) was defined as the use of anti-HER2 agents in combination with CT agents or endocrine agents; cyclin dependent kinases 4 and 6 inhibitors (CDK4/6i) or mammalian target of rapamycin (mTOR) inhibitors combined with endocrine agents; targeted agents alone; or anti-HER2 agents combined with CT agents and endocrine agents.Endocrine sensitive patients included patients with an initial diagnosis of advanced stage or more than one year after completion of adjuvant endocrine therapy (5). Primary endocrine resistance was defined as a relapse while on the first 2 years of adjuvant ET, or disease progression within the first 6 months of first-line ET for ABC, while on ET (5). Secondary endocrine resistance is defined as relapse while on adjuvant ET but after the first 2 years, or relapse within 12 months of completing adjuvant ET, or disease progression within 6 months after initiating ET for ABC, while on ET (5). Disease-free survival (DFS) was defined as the time from the date of surgery to the development of a new breast cancer event (i.e., locoregional or distant breast cancer or new primary tumor). Progression-free survival (PFS), as the primary endpoint, was defined as the time from first-line treatment to progression or death from any cause. Tumor progression was evaluated in accordance with the Response Evaluation Criteria in Solid Tumors (RECIST) version 1.1.

This study protocol was approved by each site’s ethics committee, and the requirement for informed consent was waived because this was a noninterventional study. The procedures used for data collection, saving and analyses from electronic medical records (EMRs) followed the guidelines on using real-world data to generate real-world evidence established by the National Medical Products Administration (NMPA) in China.

### Data Source

Baseline patient characteristics, treatment patterns and disease progression were extracted from patient charts, diagnostic tests, laboratory findings, and clinical notes. Patient demographics (including sex and age), TNM stage, histological type, DFS, endocrine sensitivity, ER status, PR status, HER2 status, Ki-67 index, metastatic location, number of metastatic sites, progression, and first-line treatment data were collected. After de-identification, cleaning, and standardization, the data were aggregated in the LinkDoc Breast Cancer Research Database.

### Study Population

The inclusion criteria were as follows: (1) cytologically or histologically confirmed ABC; (2) HR+ was defined as ER- and/or PR-positive; (3) at least 2 complete medical records; and (4) received first-line therapy between January 2015 and June 2019. Patients who did not receive treatment or had other primary malignancies during observation were excluded.

### Statistical Analyses

Key patient cohorts were stratified by different HER2 statuses (HER2+, HER2-, and HER2-unknown groups). Descriptive statistics of demographics, tumor characteristics and treatment patterns were performed. The Kaplan–Meier method was used to estimate PFS, which was compared across cohorts using a log-rank test. The associations between baseline patient and disease characteristics and PFS were analyzed by multivariate Cox regression. The following patient- or disease-related factors were studied as prognostic factors: first-line therapy, age, TNM stage, histological type, DFS, number of metastatic sites, metastasis sites, and Ki-67 index. For multivariate Cox regression analysis, only observations with complete information on all factors were used. No imputation was performed for missing data. The hazard ratios (HRs) with 95% confidence intervals (CIs) are presented. The significance level was set at p= 0.05. SAS version 9.4 was used for all statistical analyses.

## Results

### Demographic and Clinical Characteristics

Between January 2015 and June 2019, a total of 1072 patients with HR+ ABC who received first-line treatment were enrolled for analysis: 327 HER2+ patients, 696 HER2- patients and 49 HER2-unknown patients. The baseline characteristics of the patients are summarized in **Table 1**. Overall, the median age at diagnosis of ABC was 50.0 (43.0–56.0) years. Most patients had stage IV disease (859, 80.13%), invasive ductal carcinoma (667, 62.22%), endocrine sensitivity (409, 38.15%) and a Ki-67 index >20% (640, 59.70%). The DFS of 17.35% (186) of patients was more than 5 years, 22.11% (237) was less than 2 years, and 24.35% (261) was between 2-5 years. Of these HR+ patients, 70.43% (775) expressed both ER- and PR-positive, and 29.57% (317) expressed ER-positive or PR-positive. A total of 35.63% (382) of ABC patients had one metastatic site, and 44.50% (477) of patients had ≥2 metastatic sites. The common metastasis sites were visceral metastases (486, 45.34%), followed by soft tissue and/or lymph nodes only (327, 30.50%) and bone only (232, 21.64%).

**Table 1:** The baseline characteristics.

Characteristics, n (%)	Total (n=1072)	HER2-positive (n=327)	HER2-negative (n=696)	HER2-unknown (n=49)
**Median age at diagnosis of ABC, years (range)**	50.0 (43.0-56.0)	50.0 (44.0-56.0)	49.0 (43.0-56.0)	52.0 (45.0-61.0)
**Sex**				
Male	8 (0.75)	0	8 (1.15)	0
Female	1064 (99.25)	313 (100.00)	688 (98.85)	49 (100.00)
**Stage**				
IIIB	73 (6.81)	19 (5.81)	49 (7.04)	5 (10.20)
IIIC	140 (13.06)	42 (12.84)	87 (12.50)	11 (22.45)
IV	859 (80.13)	266 (81.35)	560 (80.46)	33 (67.35)
**Histological type**				
Invasive ductal carcinoma	667 (62.22)	212 (64.83)	422 (60.63)	33 (67.35)
Invasive lobular carcinoma	47 (4.38)	4 (1.22)	39 (5.60)	4 (8.16)
Both	217 (20.24)	66 (20.18)	144 (20.69)	7 (14.29)
Others	84 (7. 84)	28 (8.56)	52 (7.47)	4 (8.16)
Unknown/Missing	57 (5.32)	17 (5.21)	39 (5.61)	1 (2.04)
**DFS**				
>5 years	186 (17.35)	31 (9.48)	147 (21.12)	8 (16.33)
2-5 years	261 (24.35)	83 (25.38)	168 (24.14)	10 (20.41)
<2 years	237 (22.11)	86 (26.30)	137 (19.68)	14 (28.57)
Unknown/Missing	388 (36.19)	127 (38.84)	244 (35.06)	17 (34.69)
**Endocrine sensitivity**				
Endocrine sensitive*	409 (38.15)	128 (39.14)	260 (37.36)	21 (42.86)
Secondary endocrine resistance	279 (26.03)	66 (20.18)	208 (29.89)	5 (10.20)
Primary endocrine resistance	167 (15.58)	68 (20.80)	94 (13.51)	5 (10.20)
Unknown	217 (20.24)	65 (19.88)	134 (19.25)	18 (36.73)
**ER**				
Positive	1005 (93.75)	294 (89.91)	667 (95.83)	44 (89.80)
Negative	67 (6.25)	33 (10.09)	29 (4.17)	5 (10.20)
**PR**				
Positive	822 (76.68)	226 (69.11)	556 (79.89)	40 (81.63)
Negative	242 (22.57)	98 (29.97)	135 (19.40)	9 (18.37)
Unknown/Missing	8 (0.75)	3 (0.92)	5 (0.71)	0
**ER+PR**				
Both positive	755 (70.43)	193 (59.57)	527 (75.72)	35 (71.43)
Others	317 (29.57)	134 (40.43)	169 (24.28)	14 (28.57)
**Ki-67**				
≤20%	386 (36.01)	82 (25.08)	284 (40.80)	20 (40.82)
>20%	640 (59.70)	235 (71.87)	381 (54.74)	24 (48.98)
Unknown/Missing	46 (4.29)	10 (3.05)	31 (4.46)	5 (10.20)
**Number of metastatic sites**				
0	213 (19.87)	61 (18.65)	136 (19.54)	16 (32.65)
1	382 (35.63)	131 (40.06)	236 (33.91)	15 (30.61)
≥2	477 (44.50)	135 (41.28)	324 (46.55)	18 (36.73)
**Metastasis sites**				
Bone metastasis only	232 (21.64)	56 (17.13)	165 (23.71)	11 (22.45)
Soft tissue and/or lymph nodes metastasis only	327 (30.50)	93 (28.44)	217 (31.18)	17 (34.69)
Visceral metastasis	486 (45.34)	156 (47.71)	314 (45.11)	16 (32.65)

*Endocrine sensitive patients included those with an initial diagnosis at an advanced stage or more than one year after completion of adjuvant endocrine therapy.

DFS, disease-free survival; ER, estrogen receptor; PR, progesterone receptor.

Baseline demographics and clinical characteristics were generally similar among the HER2+ and HER2- groups. Compared with the HER2+ group, the HER2- group had a lower median patient age at diagnosis of ABC (49.0 vs. 50.0 years) and a higher proportion of patients who were male (1.15% vs. 0). The HER2- group had more patients with DFS > 5 years (21.12% vs. 9.48%), positive for both ER and PR (75.72% vs. 59.57%), and a Ki-67 index ≤ 20% (40.80% vs. 25.08%) than the HER2+ group.

### First-Line Treatment Strategies

Overall, as a first-line treatment, a total of 669 (62.41%) patients with HR+ ABC received CT (with or without maintenance therapy), 166 (15.49%) received ET, 226 (21.08%) received TT, and 11 (1.02%) received other therapies (**Table 2**).

**Table 2:** The most frequent treatment patterns.

Drug administration with any first-line exposure, n(%)	Total	HER2-positive	HER2-negative	HER2-unknown
	(n=1072)	(n=327)	(n=696)	(n=49)
**Chemotherapy**	669 (62.41)	93 (28.44)	536 (77.01)	40 (81.63)
Chemotherapy alone*	296 (44.25)	48 (51.61)	233 (43.47)	15 (37.50)
Chemotherapy with maintenance therapy	373 (55.75)	45 (48.39)	303 (56.53)	25 (62.50)
Chemotherapy with maintenance chemotherapy	143 (38.34)	11 (24.44)	117 (38.61)	15 (60.00)
Chemotherapy with maintenance endocrine therapy	205 (54.96)	19 (42.22)	177 (58.42)	9 (36.00)
Chemotherapy with other maintenance therapies**	25 (6.70)	15 (33.34)	9 (2.97)	1 (4.00)
**Endocrine therapy**	166 (15.49)	19 (5.81)	144 (20.69)	3 (6.12)
**Targeted therapy**	226 (21.08)	213 (65.14)	8 (1.15)	5 (10.20)
HER2-targeted therapy+chemotherapy	202 (89.38)	197 (92.49)	0	5 (100.00)
Targeted therapy+endocrine therapy	14 (6.19)	6 (2.82)	8 (100.00)	0
HER2-targeted therapy+endocrine therapy	6 (42.86)	6 (100.00)	0	0
CDK4/6i+endocrine therapy	4 (28.57)	0	4 (50.00)	0
mTOR inhibitors+endocrine therapy	4 (28.57)	0	4 (50.00)	0
Targeted therapy alone^&^	7 (3.10)	7 (3.29)	0	0
HER2-targeted therapy+chemotherapy+endocrine therapy	3 (1.33)	3 (1.40)	0	0
**Other therapy** ^#^	11 (1.02)	2 (0.61)	8 (1.15)	1 (2.05)

*Chemotherapy alone: No maintenance therapy after first-line chemotherapy due to progressive disease or toxicity.

**Chemotherapy with other maintenance therapies included targeted agents plus chemotherapy agents, targeted agents plus endocrine agents, or chemotherapy agents plus endocrine agents.

^&^Targeted therapy alone included a Bio-Thera ADC drug (a clinical study drug, BAT001), trastuzumab, and apatinib (an anti-angiogenic small molecule drug).

^#^Other therapy includes chemotherapy agents plus endocrine agents.

When examining patients with HR+/HER2+ tumors by first-line treatment after ABC diagnosis, a total of 213 (65.14%) patients received TT, 93 (28.44%) patients received CT (with or without maintenance therapy), and 19 (5.81%) patients received ET. In patients with HR+/HER2+ ABC who received CT, nearly half chose CT with maintenance therapy (45, 48.39%), and the others chose CT alone (48, 51.61%). Among those who received CT with maintenance therapy, maintenance ET (19, 42.22%) was a more frequent application than maintenance CT (11, 24.44%). HER2-targeted therapy is recommended by guidelines as standard treatment for HER2+ patients. In the present study, among patients who received TT, the majority (197, 92.49%) received HER2-targeted therapy in combination with CT agents, 6 (2.82%) received HER2-targeted therapy in combination with endocrine agents, 7 (3.29%) received targeted therapy alone, and 3 (1.40%) received HER2-targeted therapy plus CT agents plus endocrine agents.

For patients with HR+/HER2- ABC, 77.01% (536) received CT (with or without maintenance therapy), 20.69% (144) received ET and 1.15% (8) received TT. Approximately half of the HER2- patients who received first-line CT chose maintenance therapy (303, 56.53%), especially maintenance ET (177, 58.42%), followed by maintenance CT (117, 38.61%). Among patients who received TT, they underwent CDK4/6i (4, 50.00%) or mTOR inhibitors (4, 50.00%). The majority of patients with an unknown HER2 status received CT (40, 81.63%), and among them, 62.50% (25) received CT with maintenance therapy.

### Outcomes

**Figure 1:**
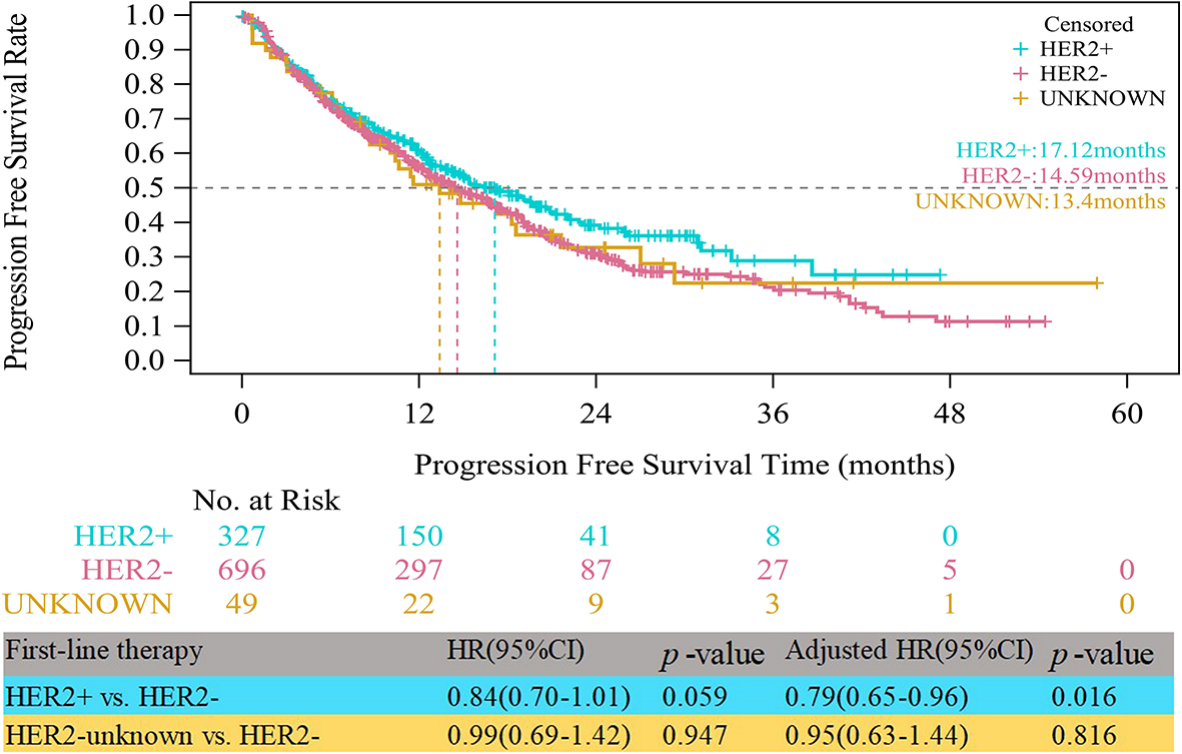
Kaplan-Meier curves of progression free survival among HER2+, HER2-, and HER2-unknown groups.

Up to the follow-up cutoff date, the median follow-up was 20.24 months overall. For all HR+ patients, the median PFS times were 17.12, 14.59 and 13.40 months in the HER2+, HER2- and HER2-unknown groups, respectively. As shown in **Figure 1**, the HER2+ group had a significantly longer median PFS than the HER2- group after multivariable adjustment (adjusted HR=0.79, 95% CI, 0.65–0.96, p=0.016). Compared with the HER2- group, the HER2-unknown group showed a lower median PFS, but the difference was not statistically significant (13.40 vs. 14.59 months, adjusted HR=0.95, 95% CI, 0.63–1.44, p=0.816).

**Figure 2:**
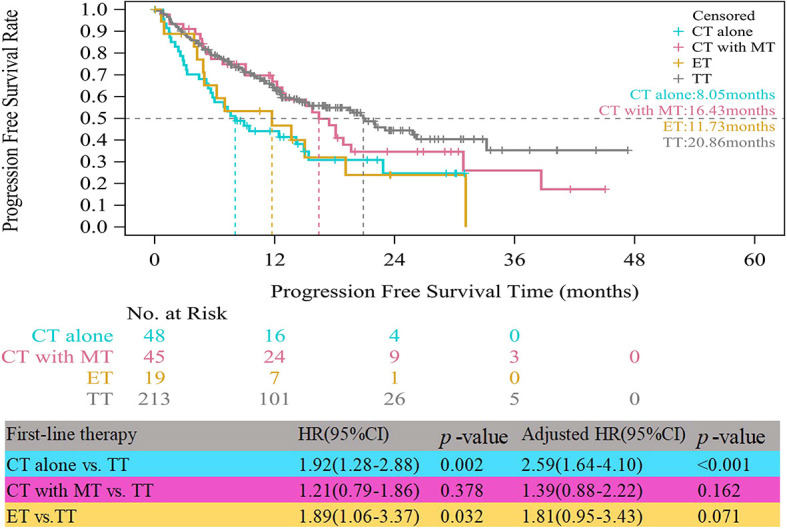
Kaplan-Meier curves of progression free survival of HR+/HER2+ patients. CT, chemotherapy; MT, maintenance therapy; ET, endocrine therapy; TT, targeted therapy.

In the HR+/HER2+ group, compared with patients who received TT, those who received CT alone experienced significantly worse median PFS (8.05 vs. 20.86 months, HR=1.92, 95% CI, 1.28–2.88, p=0.002) (**Figure 2**). The obvious difference in the median PFS between patients who received ET and those who received TT (11.73 vs. 20.86 months, HR=1.89, 95% CI, 1.06–3.37, p=0.032) was not robust because only 19 patients received ET. After multivariate analyses for PFS, the adjusted risk of disease progression in patients treated with ET was not significantly lower than that in patients treated with TT (adjusted HR=1.81; 95% CI, 0.95–3.43, p=0.071). The median PFS of patients who received CT with maintenance therapy was shorter than that of patients who received TT, but the difference was not significant (16.43 vs. 20.86 months, adjusted HR=1.39, 95% CI, 0.88–2.22, p=0.162).

**Figure 3:**
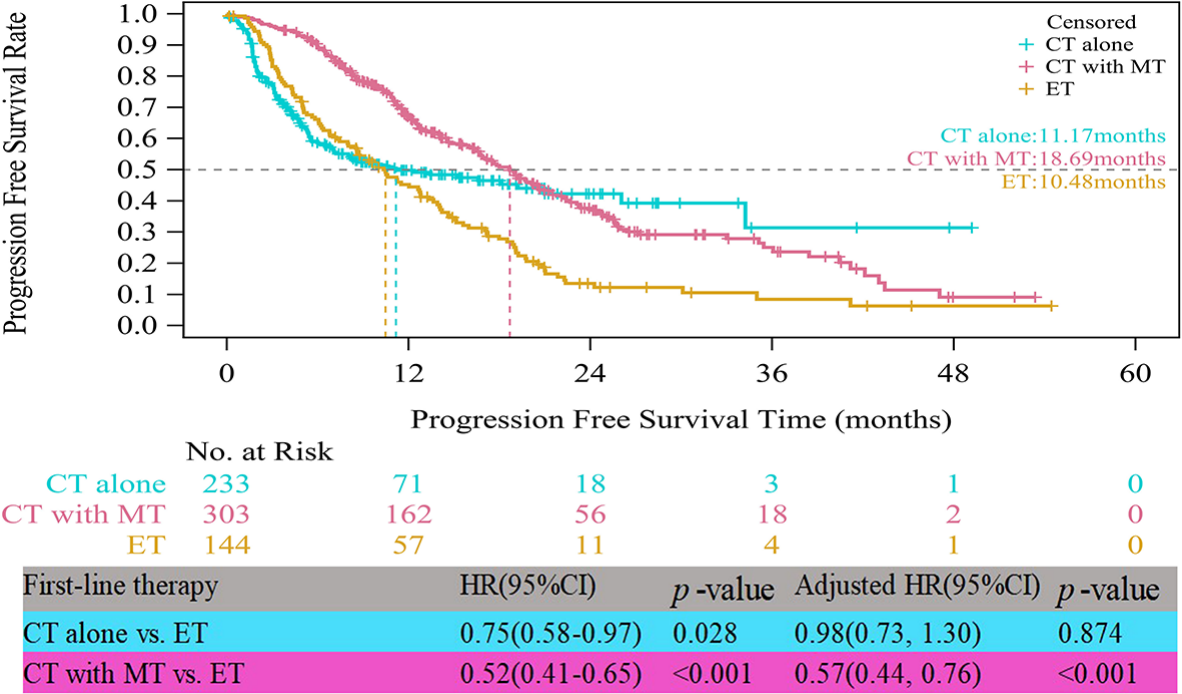
Kaplan-Meier curves of progression free survival of HR+/HER2- patients. CT, chemotherapy; MT, maintenance therapy; ET, endocrine therapy.

**Figure 4:**
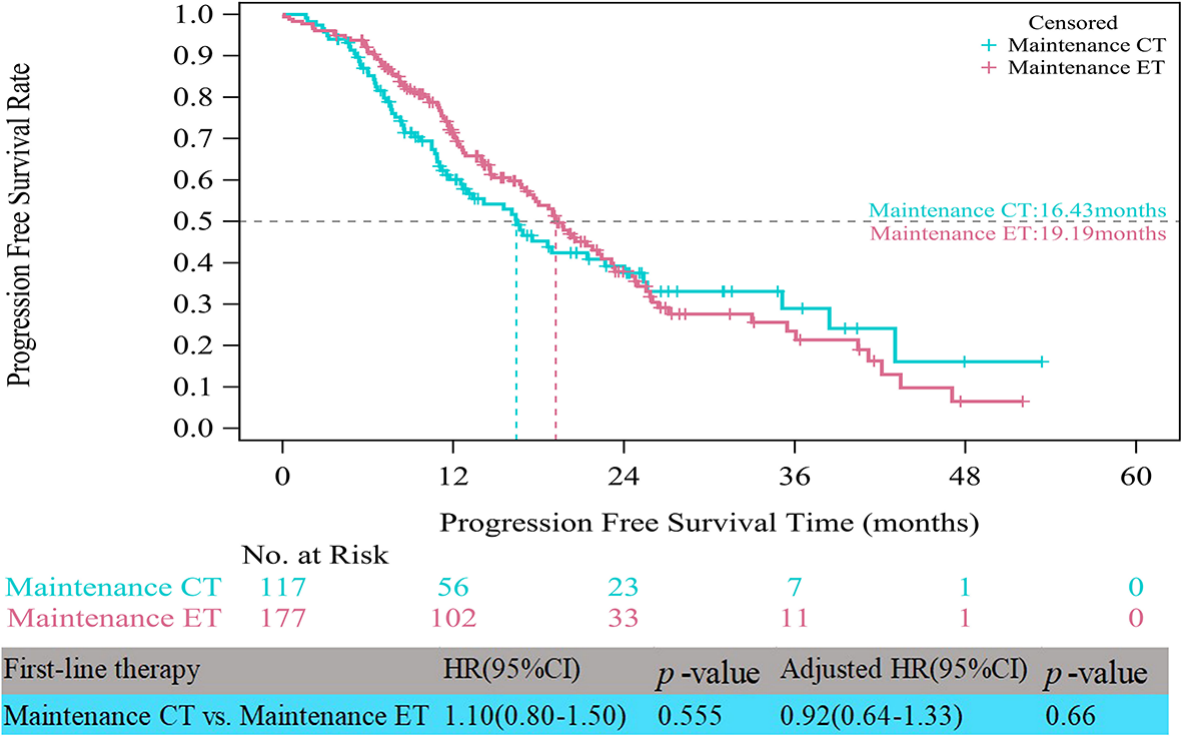
Kaplan-Meier curves of progression free survival of HR+/HER2- patients between maintenance therapies. CT, chemotherapy; ET, endocrine therapy.

In the HR+/HER2- group, the median PFS times of both patients who received CT alone (11.17 vs. 10.48 months, HR=0.75, 95% CI, 0.58–0.97, p=0.028) and those who received CT with maintenance therapy (18.69 vs. 10.48 months, HR=0.52, 95% CI, 0.41–0.65, p<0.001) were significantly prolonged compared to those who received ET (**Figure 3**). However, after multifactor analysis, we did not observe a difference in the median PFS between the ET and CT alone groups (adjusted HR=0.98, 95% CI, 0.73–1.30, p=0.874). Furthermore, we compared the median PFS between different maintenance therapy groups. **Figure 4** indicates that the median PFS of maintenance CT was slightly shorter than that of maintenance ET (16.43 vs. 19.19 months, HR=1.10, 95% CI, 0.80–1.50, p=0.555). The curve of maintenance ET declined slightly in the first two years but decreased sharply around the second year and intersected with the curve of maintenance CT.

### Clinical Prognostic Factors for PFS

To avoid confounding effects, multivariate Cox regression analysis was performed. **Table 3** indicates that according to the multivariate analysis in the HR+/HER2+ group, CT alone (adjusted HR=2.59, 95% CI, 1.64–4.10, p<0.001, reference: TT) as first-line therapy and other tumor histological types (adjusted HR=1.99, 95% CI, 1.14–3.48, p=0.016, reference: invasive ductal carcinoma) were independently associated with worse PFS. Bone metastasis only (adjusted HR=0.42, 95% CI, 0.23–0.75, p=0.004, reference: no bone metastasis only) was related to better PFS. Furthermore, in the HER2- group, CT with maintenance therapy (adjusted HR=0.57, 95% CI, 0.44–0.76, p<0.001, reference: ET), and a Ki-67 index ≤ 20% (adjusted HR=0.79, 95% CI, 0.64–0.98, p=0.033, reference: Ki-67>20%) were significant protective factors for progression.

**Table 3:** Prognostic factors of progression free survival for advanced breast cancer patients with HR-positive.

Variables	HER2-positive		HER2-negative	
	HR (95%CI)	P value	HR (95%CI)	P value
**First-line therapy**				
Targeted therapy	1			
Endocrine therapy	1.81 (0.95-3.43)	0.071	1	
Chemotherapy alone	2.59 (1.64-4.10)	<0.001	0.98 (0.73-1.30)	0.874
Chemotherapy with maintenance therapy	1.39 (0.88-2.22)	0.162	0.57 (0.44-0.76)	<0.001
**Age (years)**				
** <50**	1		1	
** ≥50**	1.06 (0.76-1.47)	0.739	0.85 (0.69-1.05)	0.140
**Stage**				
IIIB	1		1	
IIIC	0.84 (0.33-2.12)	0.713	1.08 (0.60-1.94)	0.804
IV	1.52 (0.60-3.86)	0.383	1.48 (0.80-2.77)	0.215
**Histological type**				
Invasive ductal carcinoma	1		1	
Invasive lobular carcinoma	0.95 (0.21-4.24)	0.947	1.00 (0.65-1.56)	0.992
Both	0.82 (0.50-1.36)	0.449	0.99 (0.72-1.35)	0.943
Others	1.99 (1.14-3.48)	0.016	1.04 (0.72-1.51)	0.818
**DFS (years)**				
>5	1		1	
2-5	1.07 (0.57-2.03)	0.827	1.00 (0.73-1.36)	0.979
<2	1.68 (0.77-3.65)	0.192	0.93 (0.62-1.41)	0.746
None	1.06 (0.18-6.22)	0.952	0.74 (0.42-1.30)	0.291
**Number of metastatic sites**				
≥2	1		1	
1	0.96 (0.65-1.42)	0.823	0.81 (0.63-1.03)	0.088
**Metastasis sites**				
** None**	1		1	
Bone metastasis only ** (Ref: no bone metastasis only**)	0.42 (0.23-0.75)	0.004	1.03 (0.69-1.53)	0.889
Soft tissue and/or lymph nodes metastasis only (**Ref: no soft tissue and/or lymph nodes metastasis only**)	1.28 (0.69-2.38)	0.427	1.41 (0.95-2.09)	0.092
Visceral metastasis (**Ref: no visceral metastasis**)	0.87 (0.46-1.62)	0.652	1.53 (0.98-2.38)	0.059
**Ki-67**				
>20%	1		1	
≤20%	0.92 (0.63-1.37)	0.693	0.79 (0.64-0.98)	0.033

Endocrine resistance status as a stratification factor. DFS, disease-free survival.

## Discussion

Our analysis of this multicenter, noninterventional study investigated first-line therapies and their association with clinical outcomes in patients with HR+ ABC in a real-world setting in China. This study demonstrated that the median age at ABC diagnosis in mainland Chinese patients was 50.0 years, which was nearly a decade younger than that in Western patients (15–18). In line with our findings, an epidemiological study (19) reported that the peak age of breast cancer onset in mainland China and Taiwan was approximately 45 to 49 years. The proportion of patients with visceral metastases (45.34%) was slightly higher than that in previous Western studies (20–22).

Overall, we found that first-line treatments of HR+ ABC, such as ET, CT, and HER2-specific TT, remained mainstream treatment options for the management of advanced disease, and their selection largely depended on the molecular subtype. The results showed that CT (62.41%) was widely used as an upfront therapy for HR+ patients. In the present study, a significant difference in the median PFS was found between the HER2+, and HER2- groups (HER2+ vs. HER2-, adjusted HR=0.79, 95%CI, 0.65-0.96, p=0.016). This may be due to the high proportion (65.14%) of HER2+ patients who were treated with TT, contributing to a significantly longer PFS than HER2- patients.

In patients with HR+/HER2+ ABC, we found that the most common treatment was anti-HER2 regimens plus CT-based regimens, consistent with the 2017 and 2018 Chinese Society of Clinical Oncology (CSCO) BC guideline recommendations (5, 13, 23). Compared to contemporaneous studies in Western countries (16, 24), in the present study, we observed a lower proportion (65.14%) of TT usage in Chinese HER2+ patients. This situation may be related to the high prices of targeted drugs. A previous study (25) reported that before targeted agents (containing trastuzumab) were covered by government health insurance, the estimated out-of-pocket payment by patients ranged from 3.0 to 13.1 times of the provincial average disposable annual income per capita for urban residents, and 6.2 to 27.3 times for rural residents. Until August 2017, trastuzumab was covered by government health insurance. In our study, 385 (35%) patients were included from 2015 to July 2017, and needed to pay for expensive targeted agents entirely out-of-pocket if they chose TT. However, the cost of trastuzumab has been reduced to one-third of the original cost since government health insurance coverage started. These payments were about 0.6 to 2.1 times for urban residents and 1.8 to 4.4 times for rural population (25). The financial burden for patients is still high, especially for the rural low-income population. Similar to our results, a real-world study from China suggested that 49.2% of metastatic BC patients did not receive trastuzumab as the first-line therapy (26). HER2+ ABC patients who received TT as a first-line therapy demonstrated benefits in PFS (20.86 months). In line with our finding, a phase II study suggested TT as first-line treatment in HER2+ metastatic breast cancer, with a median PFS of 19.5 months (27). Additional trials (16, 28) also showed that the addition of targeted agents to CT significantly improved PFS compared with CT alone. Similarly, observations from our study demonstrated that patients who received CT alone had significantly worse PFS than those who received TT. Moreover, there was a trend toward a longer PFS in patients treated with TT compared with those receiving CT with maintenance therapy, but there was no obvious difference between the two groups. In 45 patients treated with CT with maintenance therapy, the subsequent strategy of choosing maintenance therapy was effective in prolonging the PFS of the patients, making the difference between the two groups close. Guideline also showed there are no data to decide which is more beneficial for PFS between TT and CT with maintenance therapy in HER2+ ABC (5). Ultimately, TT should be preferred for HR+/HER2+ patients to achieve better PFS with lower toxicity.

Of note, the treatment patterns of HR+/HER2- ABC observed in the current study differed from guideline recommendations (5, 29, 30). The majority (77.01%) of patients received CT with or without maintenance therapy, in contrast to guidelines (5) that recommend ET as the standard of care in first-line settings. The top treatment pattern was CT with maintenance ET. The high proportion of patients initiating CT as first-line treatment in this paper is consistent with the result from a recent real-world study in China (31). The authors reported that 35.3% of patients with HR+/HER2- metastatic BC received ET as front-line treatment, and 17.2% of patients who had neither visceral metastasis nor progression on (neo)adjuvant ET wrongly received CT instead of ET (32). However, in Europe and America, only 25–56% of patients receive CT in the first-line metastatic setting, which is obviously different from that in China (14, 32–34). In addition, 56.53% of patients received maintenance treatment after the disease was effectively controlled by CT, and maintenance ET (58.42%) was preferred. Although maintenance treatment strategies have not been evaluated in randomized controlled trials, they have been widely applied in clinical settings (35).

We explored potential reasons for the wide usage of CT for HER2- ABC patients in routine oncology practice in China. First, a large proportion of patients with visceral metastasis (45.11%) in our cohort led to the increased use of CT. This result may be explained by the fact that the European Society for Medical Oncology (ESMO) guidelines recommend that when HR+/HER2- patients have visceral crisis, the preferred option is CT (5). Additionally, outpatient EMR data were not collected in this study. The majority of patients received ET in the outpatient department, which may have resulted in underestimating ET use in our study. Second, 13.51% of patients had primary endocrine resistance and 29.89% had secondary endocrine resistance, resulting in the inability to use endocrine drugs. Usually, patients who have endocrine resistance are not recommended to use ET for HR+ ABC by Chinese expert consensus (13). Third, compared with ET, the higher response rates and faster response associated with CT may cause clinicians to prescribe CT to patients with rapidly progressive and symptomatic disease (14, 34). This may be attributed to the vast heterogeneity in metastatic disease characteristics among these patients and the lack of clear prognostic indicators to guide physicians in identifying which patients may benefit from CT or ET. Given the generally poor prognosis of patients, physicians may use assumptions of disease response and poor overall survival rates to favor aggressive CT (33). It has been proven that patients with potentially more unfavorable characteristics, such as a higher number of metastatic sites, receive CT more often than ET (14) or receive CT first followed by endocrine maintenance treatment. Fourth, treatment costs would influence patients’ choice of agents. Reports have demonstrated that drug reimbursement policies in China strongly affect the availability of optimum systemic therapies (30). Many regimens are not covered by government health insurance, frequently resulting in prohibitively high out-of-pocket expenses for patients (3, 23). In China, a proportion of endocrine regimens and targeted regimens are not covered by insurance. Long-term administration of these drugs will be a financial burden; thus, patients’ and physicians’ decisions on whether TT or ET should be used as first-line treatment are affected by family incomes (36). Finally, based on the physician’s experience, the acceptability of CT was better than that of ET in patients. Thus, taking into account patients’ preferences, CT was more commonly used as first-line therapy.

The real-life median PFS times of ET (10.48 months) and CT alone (11.17 months) for patients with HR+/HER2- ABC in the present study were consistent with those reported in clinical trials (21). RWS showed that the PFS for ET as first-line therapy was approximately 9-12 months (18, 37), similar to that of choosing CT alone as first-line therapy. Notably, in the patient receiving induction CT followed by maintenance therapy, the PFS time almost doubled that with CT alone (18.69 months). This is most likely because maintenance therapy could have improved the prognosis of the first-line CT group (15). CT with maintenance therapy was associated with a significantly longer median PFS than ET. The results of this study indicated that maintenance therapy after first-line CT should be recommended as a first-line treatment strategy in HR+/HER2- ABC, considering its major impact on tumor progression. The rationale of maintenance therapy is based on the assumption that residual tumors contain clones that are still sensitive to one or more drugs included in the combination used as induction therapy, allowing prolonged tumor control with a decrease in side effects (38). We also compared the survival benefit among different maintenance therapies. It was interesting to note that there was no obvious difference between maintenance ET and CT. A recent meta-analysis (39) on maintenance CT concluded that time to progression was improved only in some patients and that improved PFS and overall survival were very rarely observed, whereas worsening of quality of life was the most frequent outcome. First-line maintenance ET is a considerable treatment pattern, especially when the expected benefit of continuous CT is limited or toxicity is unbearable (39).

In addition to first-line treatment patterns, we also identified significant prognostic factors for PFS. For the HR+/HER2+ subgroup, the PFS benefit was independently associated with CT with maintenance therapy and a Ki-67 index ≤ 20%. Previous studies are consistent with our results, suggesting that high Ki-67 expression was associated with worse OS and PFS (40). Bone metastasis only was identified as a protective factor, whereas CT alone was related to higher death rates in the HR+/HER2+ subgroup.

A number of limitations regarding our study should be considered when interpreting the results. First, bias was inherent because of the retrospective and observational nature of the study design. With or without available parameters, the quality of the data complicated the analyses and may have resulted in bias. Second, although data collection for PFS analysis may not have been controlled, it may closely represent real-life practice where therapies are changed almost exclusively due to progression or unacceptable side effects. Third, outpatient data were not included, which may have led to an underestimation of ET usage. Fourth, variables such as the Eastern Cooperative Oncology Group (ECOG) performance status and (neo)adjuvant therapy were not collected, and all of these variables may impact outcomes. Finally, owing to the small number of patients who received HER2-targeted therapy in combination with ET, statistical analyses could not be performed and differences between this group and HER2-targeted therapy in combination with CT were not discussed.

This real-world study demonstrated that CT (with or without maintenance therapy) remained the mainstream first-line treatment option for HR+ patients in China. Among patients with HR+/HER2+ ABC, the majority received first-line TT and experienced a PFS benefit. A high percentage of HR+/HER2- patients received CT as first-line therapy in clinical practice, not completely adhering to guidelines. There are multiple reasons for not adhering to guidelines outside of clinical trials, such as visceral metastasis, endocrine resistance, costs, response rates and patient acceptability. The results of this study indicated that CT with maintenance therapy was associated with a significantly longer median PFS than ET in HR+/HER2- ABC. Moreover, there was no obvious difference in PFS between maintenance ET and CT. Maintenance ET may be a better choice considering its lower toxicity and better quality of life.

## Data Availability Statement

The raw data supporting the conclusions of this article will be made available by the authors, without undue reservation.

## Ethics Statement

The studies involving human participants were reviewed and approved by Ethics Committee of Zhejiang Cancer Hospital, Hunan Cancer Hospital, Shandong Cancer Hospital, Anyang Tumor Hospital, Affiliated Hospital of Jiangnan University, and Baotou Cancer Hospital. The ethics committee waived the requirement of written informed consent for participation.

## Author Contributions

Conception and design: ZC, QO, and XJW. Data analysis and interpretation: YW, JW, HW, XHW, and PZ. Manuscript writing: ZC, QO, and XJW. Data collection: YW, JW, HW, XHW, PZ, JH, YBZ, WC, XS, NX, CT, HL, CW, YZ, and DR. Manuscript revision: YW, JW, HW, XHW, PZ, JH, YBZ, WC, XS, NX, CT, HL, CW, YZ, and DR. All authors contributed to the article and approved the submitted version.

## Funding

This study is supported by key research and development projects in Zhejiang Province/International cooperation technology research and development and demonstration promotion projects (2020C04012).

## Conflict of Interest

The authors declare that the research was conducted in the absence of any commercial or financial relationships that could be construed as a potential conflict of interest.

Reviewers ZY and YS declared a past co-authorship with one of the authors QO to the handling editor.

## Publisher’s Note

All claims expressed in this article are solely those of the authors and do not necessarily represent those of their affiliated organizations, or those of the publisher, the editors and the reviewers. Any product that may be evaluated in this article, or claim that may be made by its manufacturer, is not guaranteed or endorsed by the publisher.

## References

[1] Loibl S Poortmans P Morrow M Denkert C Curigliano G. Breast Cancer. Lancet (2021) 4:S0140-6736(20)32381-3. doi: 10.1016/S0140-6736(20)32381-3. 33812473

[2] Feng RM Zong YN Cao SM Xu RH. Current Cancer Situation in China: Good or Bad News From the 2018 Global Cancer Statistics? Cancer Commun (2019) 39(1):22. doi: 10.1186/s40880-019-0368-6 PMC6487510 31030667

[3] Fan L Strasser-Weippl K Li J St Louis J Finkelstein DM Yu KD. Breast Cancer in China. Lancet Oncol (2014) 15(7):e279–89. doi: 10.1016/S1470-2045(13)70567-9 24872111

[4] Yap YS Lu YS Tamura K Lee JE Ko EY Park YH. Insights Into Breast Cancer in the East vs the West: A Review. JAMA Oncol (2019) 5(10):1489–96. doi: 10.1001/jamaoncol.2019.0620 31095268

[5] Cardoso F Senkus E Costa A Papadopoulos E Aapro M André F. 4th ESO–ESMO International Consensus Guidelines for Advanced Breast Cancer (ABC 4)†. Ann Oncol (2018) 29:1634–57. doi: 10.1093/annonc/mdy192 PMC7360146 30032243

[6] Reinert T Barrios CH. Optimal Management of Hormone Receptor Positive Metastatic Breast Cancer in 2016. Ther Adv Med Oncol (2015) 7(6):304–20. doi: 10.1177/1758834015608993 PMC4622303 26557899

[7] Davie A Carter GC Rider A Pike J Lewis K Bailey A. Real-World Patient-Reported Outcomes of Women Receiving Initial Endocrine-Based Therapy for HR+/HER2- Advanced Breast Cancer in Five European Countries. BMC Cancer (2020) 20(1):855. doi: 10.1186/s12885-020-07294-2. 7. 32894087 PMC7487722

[8] Harbeck N Penault-Llorca F Cortes J Gnant M Houssami N Poortmans P. Breast Cancer. Nat Rev Dis Primers (2019) 5(1):66. doi: 10.1038/s41572-019-0111-2. 23. 31548545

[9] Cardoso F Spence D Mertz S Corneliussen-James D Sabelko K Gralow J. Global Analysis of Advanced/Metastatic Breast Cancer: Decade Report (2005-2015). Breast (2018) 39:131–8. doi: 10.1016/j.breast.2018.03.002 29679849

[10] Waks AG Winer EP. Breast Cancer Treatment: A Review. JAMA (2019) 321(3):288–300. doi: 10.1001/jama.2018.19323 30667505

[11] Giordano SH Temin S Kirshner JJ Chandarlapaty S Crews JR Davidson NE. Systemic Therapy for Patients With Advanced Human Epidermal Growth Factor Receptor 2-Positive Breast Cancer: American Society of Clinical Oncology Clinical Practice Guideline. J Clin Oncol (2014) 32:2078–99. doi: 10.1200/JCO.2013.54.0948 PMC6076031 24799465

[12] Finn RS Crown JP Lang I. The Cyclin-Dependent Kinase 4/6 Inhibitor Palbociclib in Combination With Letrozole Versus Letrozole Alone as First-Line Treatment of Oestrogen Receptor-Positive, HER2-Negative, Advanced Breast Cancer (PALOMA-1/TRIO-18): A Randomised Phase 2 Study. Lancet Oncol (2015) 16:25–35. doi: 10.1016/S1470-2045(14)71159-3 25524798

[13] Committee of Chinese Society of Clinical Oncology. Chinese Society of Clinical Oncology Breast Cancer Guideline 2017 Version 1. Beijing: People’s Medical Publishing House (2017).

[14] Miles DW Diéras V Cortés J Duenne AA Yi J O’Shaughnessy J. First-Line Bevacizumab in Combination With Chemotherapy for HER2-Negative Metastatic Breast Cancer: Pooled and Subgroup Analyses of Data From 2447 Patients. Ann Oncol (2013) 24:2773–80. doi: 10.1093/annonc/mdt276 23894038

[15] Bonotto M Gerratana L Di Maio M De Angelis C Cinausero M Moroso S. Chemotherapy Versus Endocrine Therapy as First-Line Treatment in Patients With Luminal-Like HER2-Negative Metastatic Breast Cancer: A Propensity Score Analysis. Breast (2017) 31:114–20. doi: 10.1016/j.breast.2016.10.021 27837704

[16] Kaufman PA Hurvitz SA O’Shaughnessy J Mason G Yardley DA Brufsky AM. Baseline Characteristics and First-Line Treatment Patterns in Patients With HER2-Positive Metastatic Breast Cancer in the Systhers Registry. Breast Cancer Res Treat (2021) 188(1):179–90. doi: 10.1007/s10549-021-06103-z42 33641083

[17] Tripathy D Kaufman PA Brufsky AM Mayer M Yood MU Yoo B. First-Line Treatment Patterns and Clinical Outcomes in Patients With HER2-Positive and Hormone Receptor-Positive Metastatic Breast Cancer From Registher. Oncologist (2013) 18(5):501–10. doi: 10.1634/theoncologist.2012-0414 PMC3662840 23652380

[18] Le Saux O Lardy-Cleaud A Frank S Debled M Cottu PH Pistilli B. Assessment of the Efficacy of Successive Endocrine Therapies in Hormone Receptor-Positive and HER2-Negative Metastatic Breast Cancer: A Real-Life Multicentre National Study. Eur J Cancer (2019) 118:131–41. doi: 10.1016/j.ejca.2019.06.014 31330488

[19] Sung H Rosenberg PS Chen WQ Hartman M Lim WY Chia KS. Female Breast Cancer Incidence Among Asian and Western Populations: More Similar Than Expected. J Natl Cancer Inst (2015) 107(7):djv107. doi: 10.1093/jnci/djv107 25868578 PMC4651040

[20] Mouridsen H Gershanovich M Sun Y Pérez-Carrión R Boni C Monnier A. Superior Efficacy of Letrozole Versus Tamoxifen as First-Line Therapy for Postmenopausal Women With Advanced Breast Cancer: Results of a Phase III Study of the International Letrozole Breast Cancer Group. J Clin Oncol (2001) 19(13):2596–606. doi: 10.1200/JCO.2001.19.10.2596 11352951

[21] Paridaens RJ Dirix LY Beex LV Nooij M Cameron DA Cufer T. Phase III Study Comparing Exemestane With Tamoxifen as First-Line Hormonal Treatment of Metastatic Breast Cancer in Postmenopausal Women: The European Organisation for Research and Treatment of Cancer Breast Cancer Cooperative Group. J Clin Oncol (2008) 26(30):4883–90. doi: 10.1200/JCO.2007.14.4659 PMC2652082 18794551

[22] Iwata H Masuda N Ohno S Rai Y Sato Y Ohsumi S. A Randomized, Double-Blind, Controlled Study of Exemestane Versus Anastrozole for the Fifirst-Line Treatment of Postmenopausal Japanese Women With Hormone-Receptor Positive Advanced Breast Cancer. Breast Cancer Res Treat (2013) 139(2):441–51. doi: 10.1007/s10549-013-2573-3 PMC3669502 23715630

[23] Committee of Chinese Society of Clinical Oncology. Chinese Society of Clinical Oncology Breast Cancer Guideline 2018 Version 1. Beijing: People’s Medical Publishing House (2018).

[24] Annonay M Gauquelin L Geiss R Ung M Cristol-Dalstein L Mouret-Reynier MA. Treatment and Outcomes of Older Versus Younger Women With HER2-Positive Metastatic Breast Cancer in the Real-World National ESME Database. Breast (2021) 60:138–46. doi: 10.1016/j.breast.2021.09.011 PMC8503567 34624756

[25] Diao Y Qian J Liu Y Zhou Y Wang Y Ma H. How Government Insurance Coverage Changed the Utilization and Affordability of Expensive Targeted Anti-Cancer Medicines in China: An Interrupted Time-Series Study. J Glob Health (2019) 9(2):020702. doi: 10.7189/jogh.09.020702 31673344 PMC6815654

[26] Li J Wang S Wang Y Wang X Wang H Feng J. Disparities of Trastuzumab Use in Resource-Limited or Resource-Abundant Regions and its Survival Benefit on HER2 Positive Breast Cancer: A Real-World Study From China. Oncologist (2017) 22(11):1333–8. doi: 10.1634/theoncologist.2017-0088 PMC5679829 28798274

[27] Dang C Iyengar N Datko F D'Andrea G Theodoulou M Dickler M. Phase II Study of Paclitaxel Given Once Per Week Along With Trastuzumab and Pertuzumab in Patients With Human Epidermal Growth Factor Receptor 2-Positive Metastatic Breast Cancer. J Clin Oncol (2015) 33(5):442– 447. doi: 10.1200/JCO.2014.57.1745 25547504 PMC5747317

[28] Statler AB Hobbs BP Wei W Gupta A Blake CN Nahleh ZA. Real-World Treatment Patterns and Outcomes in HR+/HER2+ Metastatic Breast Cancer Patients: A National Cancer Database Analysis. Sci Rep (2019) 9(1):18126. doi: 10.1038/s41598-019-54402-9 31792304 PMC6889133

[29] Gradishar WJ Anderson BO Balassanian R Blair SL Burstein HJ Cyr A. Nccn Guidelines Insights: Breast Cancer, Version 1.2017. J Natl Compr Canc Netw (2017) 15(4):433–51. doi: 10.6004/jnccn.2017.0044 28404755

[30] Giuliano M Schettini F Rognoni C Milani M Jerusalem G Bachelot T. Endocrine Treatment Versus Chemotherapy in Postmenopausal Women With Hormone Receptor-Positive, HER2-Negative, Metastatic Breast Cancer: A Systematic Review and Network Meta-Analysis. Lancet Oncol (2019) 20(10):1360–9. doi: 10.1016/S1470-2045(19)30420-6 31494037

[31] Yuan Y Zhang S Yan M Yin Y Song Y Jiang Z. Chemotherapy or Endocrine Therapy, First-Line Treatment for Patients With Hormone Receptor-Positive HER2-Negative Metastatic Breast Cancer in China: A Real-World Study. Ann Transl Med (2021) 9(10):831. doi: 10.21037/atm-20-8252 34164465 PMC8184482

[32] Jacquet E Lardy-Cléaud A Pistilli B Franck S Cottu P Delaloge S. Endocrine Therapy or Chemotherapy as First-Line Therapy in Hormone Receptor-Positive HER2-Negative Metastatic Breast Cancer Patients. Eur J Cancer (2018) 95:93–101. doi: 10.1016/j.ejca.2018.03.013 29655061

[33] Cobleigh M Yardley DA Brufsky AM Rugo HS Swain SM Kaufman PA. Baseline Characteristics, Treatment Patterns, and Outcomes in Patients With HER2-Positive Metastatic Breast Cancer by Hormone Receptor Status From Systhers. Clin Cancer Res (2020) 26(5):1105–13. doi: 10.1158/1078-0432.CCR-19-2350 31772121

[34] Basile D Gerratana L Corvaja C Pelizzari G Franceschin G Bertoli E. First- and Second-Line Treatment Strategies for Hormone-Receptor (HR)-Positive HER2-Negative Metastatic Breast Cancer: A Real-World Study. Breast (2021) 57:104–12. doi: 10.1016/j.breast.2021.02.015 PMC8053791 33812267

[35] Xu B Hu X Feng J Geng C Jin F Li H. Chinese Expert Consensus on the Clinical Diagnosis and Treatment of Advanced Breast Cancer (2018). Cancer (2020) 126 Suppl(16):3867–82. doi: 10.1002/cncr.32832 32710660

[36] Wu Y Han Y Yu P Ouyang Q Yan M Wang X. Endocrine Therapy for Hormone Receptor-Positive Advanced Breast Cancer: A Nation-Wide Multicenter Epidemiological Study in China. Front Oncol (2021) 10:599604. doi: 10.3389/fonc.2020.599604 33643905 PMC7905089

[37] Palumbo R Sottotetti F Quaquarini E Gambaro A Ferzi A Tagliaferri B. Patterns of Treatment and Outcome With 500-Mg Fulvestrant in Postmenopausal Women With Hormone Receptor-Positive/HER2-Negative Metastatic Breast Cancer: A Real-Life Multicenter Italian Experience. Ther Adv Med Oncol (2019) 11:1758835919833864. doi: 10.1177/1758835919833864 31210797 PMC6552357

[38] Rossi S Schinzari G Basso M Strippoli A Dadduzio V D'Argento E. Maintenance Hormonal and Chemotherapy Treatment in Metastatic Breast Cancer: A Systematic Review. Future Oncol (2016) 12(10):1299–307. doi: 10.2217/fon-2015-0065 26996100

[39] Lei W Li H Song G Zhang R Ran R Yan Y. Efficacy and Safety of Fulvestrant 500mg in Hormone-Receptor Positive Human Epidermal Receptor 2 Negative Advanced Breast Cancer: A Real-World Study in China. J Cancer (2020) 11(22):6612–22. doi: 10.7150/jca.47960 PMC7545684 33046982

[40] Lobbezoo DJ van Kampen RJ Voogd AC Dercksen MW van den Berkmortel F Smilde TJ. Prognosis of Metastatic Breast Cancer Subtypes: The Hormone Receptor/HER2-Positive Subtype Is Associated With the Most Favorable Outcome. Breast Cancer Res Treat (2013) 141(3):507–14. doi: 10.1007/s10549-013-2711-y 24104881

