# Supplementary material for: Real-World First-Line Treatment Patterns and Outcomes in Hormone Receptor-Positive Advanced Breast Cancer Patients: A Multicenter, Retrospective Study in China
Source: Front Oncol. 2022 Mar 3;12:829693. doi: 10.3389/fonc.2022.829693 (PMC8928103; doi:10.3389/fonc.2022.829693)
Supplement: Supplementary file 1 [file Table_1.docx]

Supplementary Material

# Supplementary Table 1.The patient number of each hospital

| **Hospital** | **Patients (n, %)** |
| --- | --- |
| Hunan Cancer Hospital | 389 (36.29%) |
| Shandong Cancer Hospital | 360 (33.58%) |
| Zhejiang Cancer Hospital | 127 (11.85%) |
| Anyang Tumor Hospital | 127 (11.85%) |
| Affiliated Hospital of Jiangnan University | 42 (3.92%) |
| Baotou Cancer Hospital | 27 (2.51%) |
| Total | 1072 |
